# Health Care Seeking Behavior of Persons with Acute Chagas Disease in Rural Argentina: A Qualitative View

**DOI:** 10.1155/2016/4561951

**Published:** 2016-10-18

**Authors:** Ignacio Llovet, Graciela Dinardi, Cecilia Canevari, Nahal Torabi

**Affiliations:** ^1^Departamento de Ciencias Sociales, Universidad Nacional de Lujan, Lujan, Buenos Aires, Argentina; ^2^Departamento de Posgrado, Universidad Nacional de Tres de Febrero, Caseros, Buenos Aires, Argentina; ^3^Facultad de Humanidades, Ciencias Sociales y de la Salud, Universidad Nacional de Santiago del Estero, Santiago del Estero, Argentina; ^4^Master Program of Public Health, Simon Fraser University, Burnaby, BC, Canada

## Abstract

Chagas disease (CD) is a tropical parasitic disease largely underdiagnosed and mostly asymptomatic affecting marginalized rural populations. Argentina regularly reports acute cases of CD, mostly young individuals under 14 years old. There is a void of knowledge of health care seeking behavior in subjects experiencing a CD acute condition. Early treatment of the acute case is crucial to limit subsequent development of disease. The article explores how the health outcome of persons with acute CD may be conditioned by their health care seeking behavior. The study, with a qualitative approach, was carried out in rural areas of Santiago del Estero Province, a high risk endemic region for vector transmission of CD. Narratives of 25 in-depth interviews carried out in 2005 and 2006 are analyzed identifying patterns of health care seeking behavior followed by acute cases. Through the retrospective recall of paths for diagnoses, weaknesses of disease information, knowledge at the household level, and underperformance at the provincial health care system level are detected. The misdiagnoses were a major factor in delaying a health care response. The study results expose lost opportunities for the health care system to effectively record CD acute cases.

## 1. Introduction

Detection of acute conditions of neglected infectious diseases (NIDs) is hindered by low primary health care utilization as well as low priority and quality of health care services [1]. Chagas disease (CD) is an infection caused by the parasite* Trypanosoma cruzi* and is transmitted to humans, among other ways, by an insect vector, the triatomine bug [2]. CD ranks as one of the most significant NIDs in Latin America [3]. Recent estimates suggest that more than 5.7 million people are infected with CD, mostly socially and economically vulnerable populations [4]. It produces 14,000 annual deaths in Latin America [5] and is a cause of morbidity (WHO, 2002), work disability, and increased health care costs [6]. A notable clinical consequence of* T. cruzi* is chagasic cardiomyopathy which may lead to heart failure and sudden death [7]. CD acute phase takes place two to four weeks after infection but is symptomatic in only 1% to 2% of cases. Symptoms can persist for up to four months in the absence of treatment. Typically, the acute phase develops in children under 10 years, with a 2–8% mortality rate. Diagnosis is complicated due to the nonspecific nature of the signs and symptoms and often leads to misdiagnosis [8]. According to health regulations, all confirmed CD cases require notification to the epidemiological office. Despite the success in parasite and vector control [4], Argentina regularly reports acute cases of CD, mostly young individuals [9]. Between 1996 and 2014 the number of reported acute cases ranged from a maximum of twenty-nine to a minimum of one [9, 10]. However, these figures are far from accurate. As has been underlined by official sources, most CD infection cases go unnoticed [11]. Underreporting CD figures might be explained by several factors hindering diagnosis: the largely asymptomatic nature of disease, organizational and human resources factors at the health system level [1], and social and cultural factors at the patient level [12]. In other countries this has led to question the CD prevalence figures [13]. More recent efforts are underway to review Chagas estimates [14]. Although there are estimates on patterns of health care system utilization by CD patients developed by experts [15], there is a void of knowledge of health care seeking behavior in subjects experiencing a CD acute condition [16]. In this article, through a retrospective lens we explore the latter through the health care seeking paths followed by acute cases which have been successfully diagnosed and specifically prescribed for CD treatment. By doing this, we want to better understand the factors that surround people's experience with acute CD in their quest for a diagnosis.

## 2. Health Seeking Behavior

The understanding of health care seeking behaviors is critical to determine how it affects the diagnosis and treatment of conditions. Parsons described the sick role which discusses the social role that is implied in health behavior [17]. The ill is excused to fulfill normal obligations and is not blamed for their condition. Therefore, they are not expected to heal without assistance, yet the individual holds the responsibility of seeking care and complying with medical directions. However, in the context of mainly poor rural populations that face several social and economic constraints, it is crucial to trace back the path that allows them to fulfill the main requirement of the sick role.

Broadly, the literature discusses health care seeking behaviors at the level of households and health systems. At the level of the household, health may be dependent on the availability of time, resources, priority allocated to health, cultural norms [18], and access to cash and transport [19]. At the level of the health system, issues like quality of health care delivery, that is, human resources [20], and organizational arrangements and environment influence care seekers' decisions [21, 22].

Several studies demonstrate that the cost of care or transportation to care facilities is a major barrier influencing care seeking behaviors [23, 24]. In a study done in Colombia, the most frequent path was to initially seek care at the most basic level of care which was influenced by insurance coverage and resources [15]. In the United States the absence of insurance restricted care for CD [25]. When mothers of sick children in Uganda perceived the distance to health services to be further, seeking care was delayed compared to those who perceived a shorter distance [26].

Poverty plays a role in the time it takes to seek care as well as the type of care being sought. Cost relating to care is a significant contributor to delaying care which affects diagnoses and treatment efficacy. The health care seeking behaviors of mothers with low socioeconomic status in Uganda delayed care seeking compared to those with high socioeconomic status [26]. A study on maternal health care seeking behaviors in rural Ecuador supported the hypotheses that families with lower socioeconomic status were more likely not to seek care or to treat their children's respiratory infection symptoms with home remedies [23]. Also, poverty has symbolic and moral implications. In several countries poor and rural populations are stigmatized and mistreated for being infected by CD [16]. These barriers further limit access to care and delay diagnosis in those who carry most of the disease burden.

Health system perception is an important factor that drives health care seeking behaviors. In a study done in Bolivia, rural women who followed their family's tradition of giving birth at home felt a sense of exclusion and believed the hospital would increase health complications. Others felt they would be mistreated and not informed by the staff about the procedures being done to them [27].

Latino immigrant women in the United States recognized that traditional remedies were not always the most effective but were the first line of treatment due to its lower cost. This caused a further delay in seeking care when traditional remedies were ineffective since women waited for symptoms to disappear before seeking mainstream health care [25].

Seeking care is marked by cultural and traditional elements influencing the perception of symptoms and conditions. In Mexico, bites by triatomines were considered as normal daily events and only sometimes thought to transmit disease, while others thought they had developed a resistance [28]. For other participants, reactions were considered as a result of allergy or poison [28]. Culturally normalized bites resulted in treating conditions domestically with traditional remedies. In Ghana, patients with Schistosomiasis-related symptoms were more likely to seek care only when symptoms were more severe [29].

The lack of information about symptoms, diseases, treatments, and services plays an important, yet detrimental role in health care seeking behaviors. In Bolivia, rural women were unaware of the free health services they could benefit from [26]. Similarly, one of the main barriers for Latino immigrants in Europe to access Chagas-related care was the lack of awareness of services provided to them [16]. Moreover, the fear related to CD is linked to the lack of knowledge of the disease and the treatment process. Not recognizing the severe implications of symptoms was also a significant element in delaying or not seeking health care [15, 16, 30, 31].

The review shows the different factors contributing to an array of health care seeking behaviors. Our research approaches patterns to health care from the patients' perspective, exploring the way in which families respond to the symptoms, how they address contextual limitations, and, specifically, their interactions with the health care system.

## 3. Methodology

**Figure 1:**
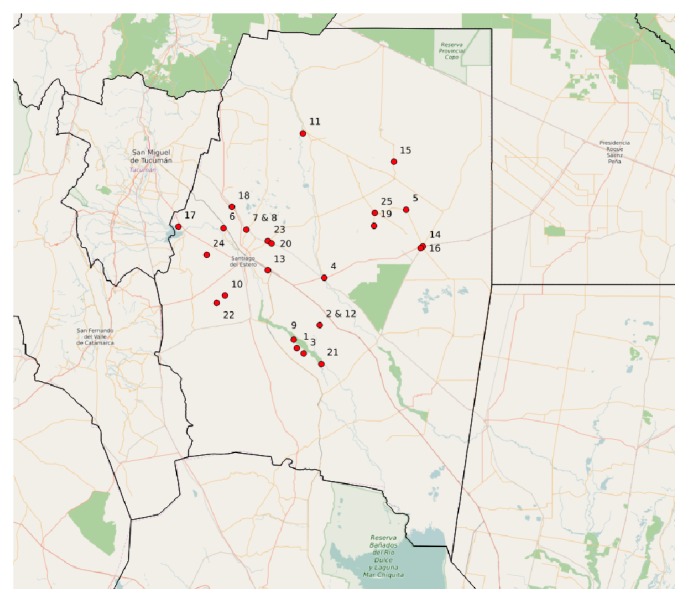
Acute cases place of residence.

**Figure 2:**
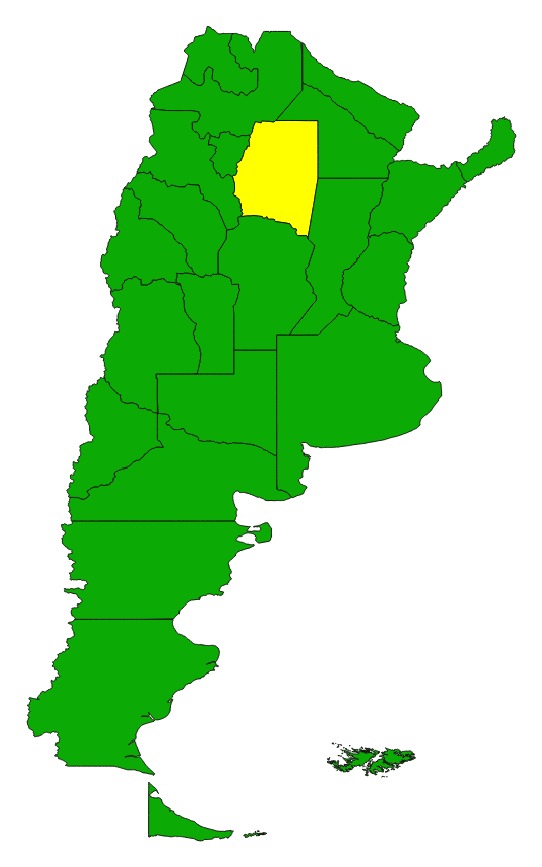
Geographical location of Santiago del Estero Province, Argentina.

An ethnographic approach of reported acute cases was applied. At the time of the study, the province of Santiago del Estero was a high risk, CD endemic [9] area with reported acute cases, a household infestation index >5%, no active surveillance, and sero-prevalence above 5% among children less than 5 years of age. The purpose was to collect data regarding the experience of the acute case's family from the time of the outbreak to the sero-positive laboratory result and to find care seeking patterns in these processes. Twenty-five acute cases out of forty reported in 2003, 2004, and 2005 [32, 33] were interviewed, 14 females and 11 males, that were registered by the health provincial system (Table 1). As minors, most of the acute cases were represented by a care taker, who was the respondent in the interview. The in-depth interviews had an average length of three hours. As twenty-three out of the twenty-five subjects were under 15 years old, the retrospective information collected during the interview was provided by close relatives, usually the mother. Both long and short term retrospective recall can provide an accurate report of occurrence of health related events [34]. Also, family members playing a caring role, particularly mothers, are in position to provide accurate proxy reports of disease and symptoms [35]. Distance from the provincial capital city, Santiago del Estero, to acute cases' houses ranged from 12 to 300 km, in villages distributed across 11* departamentos*, a subprovincial administrative level (Figures 1 and 2). Most of the acute cases were located in rural areas of particularly difficult access: unpaved and dirt roads, narrow and fainting trails, and with no means of public transportation. The average time between the outbreak and the interview was 11 months, ranging from 3 to 22 months. Interview data was processed with NUDI.ST software.

## 4. Findings

### 4.1. Acute Cases

Mostly, the study population was from poor rural families with minimal access to water and electricity. Most homes were made out of mud, with cracks and crevices that were ideal to accommodate* T. infestans* (known in Argentina as* vinchuca*). Homes that had a bathroom, access to electricity, and easy access to water were considered as having standard living conditions (Table 1). Those who had health posts in their area mostly had irregular or minimal attendance of health practitioners or did not have the resources to deal with the health problems presented by the families. The most common symptom of the acute cases was a swollen eye (17 out of 25) which is a clinical symptom specific to CD. At times, the main condition was accompanied by other symptoms including fever, red eyes, or body pain. Some families were unaware that these symptoms could be a result of CD and these nonspecific symptoms led them to think they acquired other conditions, which influenced their method of care. Others were not aware of the existence of CD and how it was caused; there were few families who were knowledgeable about the transmission of the disease. Direct parasitological methods, that is, micromethod in children and Strout method in adults, were used to diagnose* Trypanosoma cruzi* infection at the acute phase. There were five different health care seeking behavior pathways the families took in order to finally get the subject diagnosed with acute CD.

### 4.2. Pathways to Care

In 2000, 51 hospitals and 254 primary health care units (PHCUs) organized with different levels of complexity were part of the provincial health care system [36]. At the bottom, the PHCUs which were mostly located in rural areas offered outpatient services. Although some of them were staffed with physicians, many were looked after by auxiliary nurses with basic training. At the top of the system, in the capital city, there was a referral hospital (hereafter, General Hospital, GH). It had a specialized center for CD diagnosis and treatment as well as a laboratory equipped for specific studies. In an intermediate position, smaller and lower complexity hospitals, with permanent on-call doctors, delivered basic health services.

At the onset of symptoms, subjects searched for care at different levels of the system. In this endeavor, they followed a variety of paths ending with a sero-positivity diagnosis. These paths had a recognizable pattern.

### 4.3. One Step

Six out of the 25 acute cases studied were diagnosed in one step. These six individuals are classified into three distinct methods of health care seeking behaviors. One individual directly attended the hospital due to the absence of a primary health care unit (PHCU), four dismissed the local PHCU as the first line of care, and one was taken to GH by health personnel working in the field where they were diagnosed.

There are many reasons for which subjects dismissed the PHCU as the lack of consistency in the schedule, “*E: but it's nothing but a tale because sometimes the nurse is there and sometimes he isn't (…) because in this zone we are disadvantaged …*” (Subject 11); even with a stable schedule, patients had no choice but to seek care at the hospital when doctors attended the post infrequently, “*E: Yes there is a health post here… (Doesn't the doctor come?) E: Yes, on Mondays… (And do you go for consultations there?)…E: If it isn't in the morning, if you go in the afternoon you need to go to Santiago*.” (Subject 10). Some health posts only had nurses, and local residents could not reach a doctor unless they went to the hospital. In some cases, it had been so long since a doctor had attended the health post they did not expect one to be present, “*(Do you have a health post?) E: Yes…nothing but a nurse. (Doctors do not come?) E: No, sometimes from Medellín…every two months*.” (Subject 3). Those who had access to a primary health care unit were not always able to be cared for due to policies regarding the need for a doctor's reference: “*(You don't go to the health post? E: Yes, sometimes…but you need a doctor's referral, otherwise they will not attend to you)*” (Subject 20).

In addition to the limitations mentioned in these four cases, the health care seeking behaviors were driven by the proximity to the hospital. The family of the fifth case was an exception as they chose to be transferred to the capital city (135 km) as a relative lived there. Obtaining a diagnosis was not free of complications; an 11-year-old boy was diagnosed with an eye infection. Immediately, upon his father's insistence, a second test was carried out in the same hospital where CD was diagnosed.

### 4.4. Two Steps

Fourteen out of the 25 cases went through two steps to get diagnosed. As the first line of care, twelve individuals attended a hospital, one visited a PHCU, and one consulted a healer. All these cases ended their pathway in GH, where they were diagnosed and/or treated.

From the total of the 12 individuals that made it to different hospitals for their first consult, 5 were diagnosed with CD and were transferred to GH for treatment, six cases were transferred directly to GH without diagnosis, with the “presumption” of CD, and one attended the GH not by transfer but guided by the informal advice of a nurse.

Of the 12 cases, 10 travelled between 3 and 25 km and attended hospitals located outside their area of residence, while two attended a hospital in their area of residence. Among those who travelled outside of their locality, three did not have a PHCU in their area of residence and went to nearby hospitals. In the rest of the seven cases, the families had a PHCU in their locality; however these were dismissed, as their first consult. They preferred to attend the hospital, even when this choice implied heavier costs in terms of time and money. Five of the cases had a negative view on the care provided by the PHCU in regard to the lack of supplies and the lack of consistency in care “*yes, (…) the post is for decoration because we don't have medications, nothing…*” (Subject 1) “*E: Because it does not have anything. They only weigh her…*” (Subject 15). “*And now the nurse is going on leave and we no longer have one… (a post) (…) until January*.” (Subject 6).

A sixth individual, although satisfied with the closest PHCU in another village because it was a source of milk and vaccination, and doctors attended regularly, decided to attend a hospital.

The selection of the health establishment seemed to be determined by its proximity.

#### 4.4.1. The PHCU as the First Line of Care

The only subject that chose the PHCU as first line of care lived in a small area with difficult access, 13 km away. Faced with the symptoms, the mother attended the post where the acute case was transferred to GH, 127 km away, with the presumption of CD. There, the individual was diagnosed and treated.

#### 4.4.2. The “Healer” as the First Line of Care

Distrusting the health system, this subject sought care from a healer who had “cured” one of the adults in the family from CD and had also treated symptoms of parasitic infections in children. The healer suggested transferring the girl to GH: “*E: He is a healer in the countryside that heals…then I took her there, and he tells me (…) this is a vinchuca bite. He tells me you have to take her to GH (…) and then I (…) took her. It's already advanced, he tells me, I will not be able to cure her for you*.” (Subject 17).

#### 4.4.3. Misdiagnosis

Of the 14 acute cases, the four that received a misdiagnosis attended diverse hospitals. These wrongfully diagnosed cases returned to their homes and with the persistence of the acute symptoms went back for another consult. Three were then referred to GH with a presumption of CD. The fourth case, unsatisfied with a second diagnosis, attended GH by own decision, without referral.

### 4.5. Three Steps

Three out of the 25 studied subjects went through three health steps. The first step for two of them was to attend the PHCU while the remaining was unidentifiable. In the second line of care, the cases attended different hospitals, until they ended their pathway in GH. In this search, they were all misdiagnosed.

The choice of the health post was driven by its proximity. In one of the cases, the urgency to respond to the symptoms of the acute case prevailed over the negative view of the care offered by the PHCU. The closest hospital was located at 12 km: “*E: No, because here they don't want to medicate, they don't want to examine. (…) If you go ask for a remedy they say there is not any, they don't want to give it to you, so you have to travel by force*” (Subject 7).

The other case that attended the PHCU belonged to a small rural area without any services. For this family, the closest PHCU was located at 19 km and offered unsatisfactory care.

#### 4.5.1. Misdiagnoses

The three subjects received false diagnoses in their first consult and returned home: “*E: She became worse, she didn't sleep and at night she would cry and cry, on top of that she had fever… From there, we started to get worried and from there I took her to (Hospital), but they gave her drops, but for the eye*.” (Subject 7). Similarly to other misdiagnosis, the persistence and the worsening of symptoms was a source of worry for the family, where the acute cases felt an impulse to search for other answers:* E: And she started to (…) have inflammation in the eye, and since I didn't know what Chagas was… (…), and I took her to the doctor and they gave me a lot of drops to give her. (And what did the doctor tell you?) E: He said that it was conjunctivitis, and from there a nurse told me: “No, I don't think it's conjunctivitis because it needs to be in both eyes*.” (Subject 2).

### 4.6. Four Steps

Subject 12 went through four health instances before being diagnosed and treated at GH. He was transferred twice, always without a diagnosis despite having done tests, until reaching GH.

At first, the mother attributed the inflammation of the eye erroneously to an accident involving a nail. She treated him with edible oil, but once the symptoms worsened she searched for help:* E: Well, there was the health train that came from Buenos Aires, (…) here to the village (…) I took him (…) and there they did nothing but transfer me urgently to “F” (zonal hospital), because they thought that…since I had told them it was from a nail…From “F” they transferred me urgently to Santiago, to hospital “N”*. From the zonal hospital located 52 km from their place of residence, the acute case was transferred to the hospital “N” located 122 km away, where he stayed for two weeks. The mother, due to past family history, was knowledgeable about CD. During her interactions with health personnel she communicated her suspicion that grew stronger as the days passed and the symptoms persisted: “*E: I told her, look doctor, please I ask you to do an analysis of Chagas”. Because I had told her that I had vinchucas (…) and I tell her “there has already been a case doctor, a Chagas problem there too and the girl was like him, like this.*”

Since the boy did not get better, the mother requested the director of the hospital to transfer him to GH: “*(…) Finally, I had to get angry; I had no other choice (…). I was going to sign and I was going to take him because I saw no improvement, I was going to take him somewhere else, look for other doctors, my intentions were to take him to GH….*” The director authorized the transfer to GH in an ambulance accompanied by a nurse and the doctor who had treated him. Upon arriving to GH, and based on his clinical manifestations, CD was diagnosed.

### 4.7. Five Steps

This individual went through three different hospitals and a healer and ended up being diagnosed with CD in GH.

The acute symptom initially appeared as general inflammation of the body, discomfort, crying, and sleeping problems. The parents dismissed the PHCU in their zone where they had not been treated well.

The boy was misdiagnosed three times. The suspicion of child physical abuse was the reason this individual had to pass through five steps of seeking diagnosis. Each hospital believed that the symptoms were caused by burns and did not believe the parents. The father and the mother always attended the health centers together: “*E1: They thought we had burned him. (…) And we said that it was not a burn. (…) They… gave him some drops so it doesn't burn, you see. E2: They said the same thing there at (other hospital), that it was a burn. I say to them, no sir*.” (Subject 18).

In hospital “P”, the boy received a diagnosis for a throat condition, for which he was prescribed an antibiotic treatment: “*E: He started to swell up (…) and he cried all night, we took him and the doctors *didn't* give him anything. He cried until the next day. And from there I took him to (other hospital) again and the doctors gave me medication because they said it's a sore throat*.” (Subject 18).

Faced with persistent symptoms, the parents consulted a healer, who determined that the boy suffered from a “scare,” which he had treated successfully before, and thereafter, the discomfort and crying disappeared. However, within a few days, the boy presented with a lesion in the arm, which prompted a new consult at the hospital “P”. The doctors wanted to keep the boy admitted in “P” but the parents decided to take him to “L” hospital. The doctors from “L” confirmed that it was a burn, although the parents insisted that the boy had not gotten burned.

The case was admitted to hospital “L”, where they did numerous examinations. Finally, he was transferred to GH where he received diagnosis for CD and treatment. The parents completed all the follow-up controls.

## 5. Discussion

The trajectories of the 25 subjects bring to light active health care seeking behaviors, of which there are five identifiable pathways. These pathways include PHCUs, different levels of hospitals, the referral hospital (GH), and natural healers. As the number of steps increased, so did the cost of the transfers that came with the search and the disruption that took place in the everyday lives of the families. However, this did not prevent seeking care, in contrast to Oxaal & Cook's findings [17]. Attending health centers was not delayed either, despite the distances that had to be travelled, as was the case in Rutebemberwa et al. [26].

Various studies have shown that payments for medical services cause a differentiation in health care seeking [15, 23]. In Argentina, this restriction is not relevant considering that access to public health care services is free.

The socioeconomic condition of the study population is mainly homogenous. Although there are three families with more stable incomes, the majority have precarious financial means with incomes consisting of governmental cash transfers, remittances from family members, and occasional paid work. The insecurity of income explains the predominance of substandard dwellings and with that a favorable habitat for* T. infestans*. In contrast to what is noted in other studies [16], the relative socioeconomic conditions of the acute cases studied did not influence the number of steps the households took to seek care.

The difficulty to diagnose the disease during the acute phase is shown in various studies and countries due to the heterogeneity of symptoms that tend to overlap with other diseases. In accordance with the guidelines for patients' care, all specific and nonspecific symptoms in endemic zones should be presumed CD. Specific symptoms are present in only 5% of acute cases [11]. In the population studied, there were specific clinical symptoms in 17 of the 25 individuals. Nevertheless, ten cases received a misdiagnosis, eight of which had specific symptoms. Misdiagnoses played a key role in prolonging care seeking.

The weak surveillance systems hindered the capacity of catching new cases. According to a study conducted in Venezuela “the acute-phase patient is only detected by chance or by extending sero-parasitologic diagnosis to people living in the vicinity of an identified acute case” [37]. Coincidently, in the study population, only one case was detected as a result of a blood extraction campaign at the population level.

As the first symptoms appeared, in many cases seeking care was oriented by the expectation of an effective answer from the system. In 12 cases, the first decision consisted of not attending the PHCU due to a negative view on the care they offer, which is consistent with other studies [27]. Despite a negative opinion, three subjects decided to attend the PHCU due to the severity of the symptoms.

Minneman et al.'s study on the Latino immigrant population in the United States [24] reports a widespread use of home remedies. In the study population, only two individuals used home remedies as the first response to symptoms, which delayed seeking care in the health system. The role of healers was also very small, for those who resorted to one as the first line of care.

Similar to the findings reported in Mexico [28], the study subjects were familiar with triatomine bugs but were not able to connect it with the disease, nor did they have the necessary information for its prevention. Only in few cases where relatives or neighbors had experienced the disease were they aware of CD.

## 6. Conclusions

The reported acute cases' pathways show the effort and the perseverance of families to overcome obstacles presented by vulnerable social and economic conditions. They played out the sick role model put forward by Parsons.

Most individuals with acute CD reached diagnosis after going through several health services. From the patient perspective, that process was costly in material and symbolic terms. Poor information on CD and its symptoms was a factor that conditioned the way families sought care for their health concerns. On the system's side, health care delivery was inefficient. The misdiagnoses were a major factor in delaying a proper health care response.

This study stresses the health system's weakness for the detection of acute cases and consequently its epidemiological registry. More effort is needed to strengthen medical training to diagnose CD conditions in endemic areas. The effects of a better diagnostic capacity will be reflected in more accurate CD statistics.

## Figures and Tables

**Table 1:** Acute cases.

Case	Age	Sex	Caretaker	Primary health care unit in the vicinity (yes/no)	Living conditions (standard/substandard)	Health care seeking behavior pathway	Misdiagnosis	Principal clinical symptom
1	2	M	Mother	Y	Standard	2	Y	Swollen eye
2	2	F	Mother	N	Sub	3	Y	Swollen eye
3	5	F	Grandmother	Y	Sub	1	N	Swollen leg
4	15	M	Mother	Y	Sub	2	N	Swollen eye
5	2	F	Mother	Y	Sub	2	N	Swollen face
6	1	F	Mother	Y	Sub	2	Y	Swollen eye
7	1	F	Mother	Y	Sub	3	Y	Swollen eye
8	7	M	Mother	Y	Sub	3	Y	Renal problems
9	13	F	Mother	N	Standard	2	N	Swollen eye
10	7	F	Mother	Y	Sub	1	N	Swollen eye
11	3	F	Mother	N	Sub	1	N	Swollen eye
12	12	M	Mother	N	Sub	4	Y	Swollen eye
13	9	M	Mother and father	N	Sub	2	N	Swollen eye
14	3	F	Mother	N	Sub	2	N	Swollen eye
15	4 mo.	F	Mother	Y	Sub	2	N	Purple skin
16	11	M	Mother	Y	Sub	2	N	Swollen eye
17	3	F	Mother	N	Standard	2	N	Swollen eye
18	1	M	Father	Y	Sub	5	Y	Persistent crying
19	8	M	Father	Y	Sub	2	N	Fever
20	26	M	Himself	Y	Sub	1	N	Swollen face and neck
21	11	M	Father	Y	Sub	1	Y	Swollen eye
22	12	F	Mother	N	Sub	2	N	Swollen eye
23	6	F	Mother	Y	Standard	2	Y	Swollen eye
24	8	F	Mother	Y	Sub	2	Y	Swollen eye
25	5	M	Mother	Y	Sub	1	N	Body swelling

## References

[1] Unger J.-P., De Paepe P., Green A. (2003). A code of best practice for disease control programmes to avoid damaging health care services in developing countries. *International Journal of Health Planning and Management*.

[2] Prata A. (2001). Clinical and epidemiological aspects of Chagas disease. *The Lancet Infectious Diseases*.

[3] Hotez P. J., Molyneux D. H., Fenwick A. (2007). Control of neglected tropical diseases. *The New England Journal of Medicine*.

[4] World Health Organization (2015). Chagas disease in Latin America: an epidemiological update based on 2010 estimates. *The Weekly Epidemiological Record*.

[5] McKee M. (2010). The World Health Report 2000: 10 years on. *Health Policy and Planning*.

[6] Dias J. C. P. (2007). Globalization, inequity and Chagas disease. *Cadernos de Saude Publica*.

[7] Rassi A. Jr., Rassi A., Little W. C. (2000). Chagas' heart disease. *Clinical Cardiology*.

[8] Lugones H. S. (2001). *Enfermedad de Chagas. Diagnostico de Su Faz Aguda*.

[9] Ministerio de Salud de la Nacion (2011). Boletin integrado de vigilancia. *Secretaría de Promoción y Programas Sanitarios*.

[10] Ministerio de Salud—OPS (2015). *Argentina 2015*.

[11] Ministerio de Salud de la Nacion (2012). *Guías Para La Atencion Al Paciente Infectado Con Trypanosoma cruzi*.

[12] Franco-Paredes C., Von A., Hidron A. (2007). Chagas disease: an impediment in achieving the Millennium Development Goals in Latin America. *BMC International Health and Human Rights*.

[13] Attaran A. (2006). Chagas' disease in Mexico. *The Lancet*.

[14] Hotez P. J., Dumonteil E., Woc-Colburn L. (2012). Chagas disease: ‘The New HIV/AIDS of the Americas’. *PLoS Neglected Tropical Diseases*.

[15] Castillo-Riquelme M., Guhl F., Turriago B. (2008). The costs of preventing and treating Chagas disease in Colombia. *PLoS Neglected Tropical Diseases*.

[16] Ventura-Garcia L., Roura M., Pell C. (2013). Socio-cultural aspects of chagas disease: a systematic review of qualitative research. *PLoS Neglected Tropical Diseases*.

[17] Parsons T. (1975). The sick role and the role of the physician reconsidered. *Health and Society*.

[18] Oxaal Z., Cook S. (1998). Health and poverty gender analysis. *BRIDGE*.

[19] Manderson L. (1998). Applying medical anthropology in the control of infectious disease. *Tropical Medicine and International Health*.

[20] Hongoro C., McPake B. (2004). How to bridge the gap in human resources for health. *The Lancet*.

[21] Hall J. J., Taylor R. (2003). Health for all beyond 2000: the demise of the Alma-Ata declaration and primary health care in developing countries. *Medical Journal of Australia*.

[22] MacKian S. (2003). *A Review of Health Seeking Behaviour: Problems and Prospects*.

[23] Luque J. S., Whiteford L. M., Tobin G. A. (2008). Maternal recognition and health care-seeking behavior for acute respiratory infection in children in a rural ecuadorian county. *Maternal and Child Health Journal*.

[24] Beiersmann C., Sanou A., Wladarsch E., De Allegri M., Kouyaté B., Müller O. (2007). Malaria in rural Burkina Faso: local illness concepts, patterns of traditional treatment and influence on health-seeking behaviour. *Malaria Journal*.

[25] Minneman R. M., Hennink M. M., Nicholls A. (2012). Barriers to testing and treatment for Chagas disease among Latino immigrants in Georgia. *Journal of Parasitology Research*.

[26] Rutebemberwa E., Kallander K., Tomson G., Peterson S., Pariyo G. (2009). Determinants of delay in care-seeking for febrile children in eastern Uganda. *Tropical Medicine & International Health*.

[27] Rööst M., Jonsson C., Liljestrand J., Essén B. (2009). Social differentiation and embodied dispositions: a qualitative study of maternal care-seeking behaviour for near-miss morbidity in Bolivia. *Reproductive Health*.

[28] Valdez-Tah A., Huicochea-Gómez L., Ortega-Canto J., Nazar-Beutelspacher A., Ramsey J. M. (2015). Social representations and practices towards triatomines and chagas disease in Calakmul, México. *PLoS ONE*.

[29] Danso-Appiah A., Stolk W. A., Bosompem K. M. (2010). Health seeking behaviour and utilization of health facilities for schistosomiasis-related symptoms in ghana. *PLoS Neglected Tropical Diseases*.

[30] Donovan S. D., Stevens M., Sanogo K., Masroor N., Bearman G. (2014). Knowledge and perceptions of Chagas disease in a rural Honduran community. *Rural and Remote Health*.

[31] Wang J., Fei Y., Shen H., Xu B. (2008). Gender difference in knowledge of tuberculosis and associated health-care seeking behaviors: a cross-sectional study in a rural area of China. *BMC Public Health*.

[32] Ministerio de Salud—OPS (2005). *Indicadores Básicos. Argentina 2005*.

[33] Ministerio de Salud—OPS *Indicadores Básicos: Argentina 2006*.

[34] Beckett M., Da Vanzo J., Sastry N., Panis C., Peterson C. (2001). The quality of retrospective data: an examination of long-term recall in a developing country. *Journal of Human Resources*.

[35] Klinkenberg M., Smit J. H., Deeg D. J. H., Willems D. L., Onwuteaka-Philipsen B. D., van der Wal G. (2003). Proxy reporting in after-death interviews: the use of proxy respondents in retrospective assessment of chronic diseases and symptom burden in the terminal phase of life. *Palliative Medicine*.

[36] Dirección de Estadísticas e Información en Salud (DEIS) (2000). *Guía de Establecimientos de la Salud*.

[37] Añez N., Carrasco H., Parada H. (1999). Acute Chagas' disease in western Venezuela: a clinical, seroparasitologic, and epidemiologic study. *The American Journal of Tropical Medicine and Hygiene*.

